# Control of Nitrogen Inhomogeneities in Type-I and Type-II GaAsSbN Superlattices for Solar Cell Devices

**DOI:** 10.3390/nano9040623

**Published:** 2019-04-17

**Authors:** Nazaret Ruiz, Verónica Braza, Alicia Gonzalo, Daniel Fernández, Teresa Ben, Sara Flores, José María Ulloa, David González

**Affiliations:** 1 University Research Institute on Electron Microscopy & Materials, (IMEYMAT) Universidad de Cádiz, 11510 Puerto Real (Cádiz), Spain; veronica.braza@uca.es (V.B.); teresa.ben@uca.es (T.B.); sara.flores@uca.es (S.F.); david.gonzalez@uca.es (D.G.); 2 Institute for Systems based on Optoelectronics and Microtechnology (ISOM), Universidad Politécnica de Madrid, Avda. Complutense 30, 28040 Madrid, Spain; agonzalo@isom.upm.es (A.G.); jmulloa@isom.upm.es (J.M.U.)

**Keywords:** superlattice, type-I and –II, solar cells, dilute nitride, TEM

## Abstract

Superlattice structures (SLs) with type-II (GaAsSb/GaAsN) and -I (GaAsSbN/GaAs) band alignments have received a great deal of attention for multijunction solar cell (MJSC) applications, as they present a strongly intensified luminescence and a significant external quantum efficiency (EQE), with respect to the GaAsSbN bulk layers. Despite the difficulties in characterizing the distribution of N in dilute III-V nitride alloys, in this work we have obtained N-compositional mappings before and after rapid thermal annealing (RTA) in both types of structures, by using a recent methodology based on the treatment of different scanning transmission electron microscopy (STEM) imaging configurations. Texture analysis by gray level co-occurrence matrixes (GLCM) and the measurement of the degree of clustering are used to compare and evaluate the compositional inhomogeneities of N. Comparison with the Sb maps shows that there is no spatial correlation between the N and Sb distributions. Our results reveal that a better homogeneity of N is obtained in type-I SLs, but at the expense of a higher tendency of Sb agglomeration, and the opposite occurs in type-II SLs. The RTA treatments improve the uniformity of N and Sb in both designs, with the annealed sample of type-II SLs being the most balanced structure for MJSCs.

## 1. Introduction

Nanostructured solar cells [1] offer a perfect strategy to increase the efficiency using less material in device fabrication. In this sense, ultra-thin solar cells [2], dye-sensitized solar cells [3], quantum-dots-sensitized solar cells [1], small molecule organic solar cells [4], or perovskite solar cells [5] have demonstrated a great potential to convert solar energy into electric energy at a low cost. At the other extreme, tandem multijunction solar cells (MJSCs) have made a breakthrough in the field of modern photovoltaic research, holding the records of conversion efficiencies under concentration with a significant difference over all the rest of the technologies [6]. Their higher production costs can be made up of the combination of ultra-high-efficiency solar cells (SCs) with optical concentration. In this field, diluted III-V nitride alloys have attracted much attention after the two consecutive world efficiency records, set by Solar Junction CA, using a new GaInNAs sub-cell in combination with Sb as surfactant [7]. In a step forward, dilute GaAsSbN nitride has been proposed as an attractive alternative sub-cell in the range 0.9 < E_g_ < 1.3 eV, as it offers the possibility of achieving a longer wavelength, with a lower N concentration compared to GaInNAs [8,9]. In this sense, type-II (GaAsSb/GaAsN) and type-I (GaAsSbN/GaAs) superlattices (SLs) have recently been reported to significantly improve the external quantum efficiency under photovoltaic conditions, with regard to bulk layers of equivalent thickness [10,11]. Like the other dilute nitrides, the extremely low solubility of N in these alloys (the range of dilute nitrogen is <5%) suggests that high-quality III-V-N structures, suitable for minority carrier devices, may be difficult to grow. Composition fluctuations and morphological instabilities occur even if this type of dilute nitride alloy is grown under metastable conditions [12,13,14]. Certainly, the presence of these inhomogeneities strongly influences optical properties, as they create potential random fluctuations, which amplify the electron density of the states [15,16].

The introduction of a fourth element in GaAsN alloys with a larger radius than GaAs elements, such as In or Sb, facilitates the growth of high-quality layers, as it compensates for the tensile effect of N [17]. However, in the case of GaInAsN, the need to incorporate more than 2% N and 25% In to reach the desired wavelength range causes large fluctuations in the composition and roughness of the interface, which decreases structural quality [18,19,20]. In the case of GaAsSbN, the high improvement of the photoluminescence (PL) by the incorporation of Sb has been generally attributed to a more homogeneous distribution of N, and to a reduction in the formation of N complexes [21,22,23]. However, other authors propose that, in fact, the GaAsSbN alloy presents the largest compositional inhomogeneity among the dilute nitrides, and the reason for PL enhancement is that the excitons are more efficiently trapped in these alloy fluctuations [24].

On the other hand, it is widely accepted that the rapid thermal annealing (RTA) process improves the homogeneity of N within dilute nitride layers, and this is based on the increase of the PL intensity, decreasing its full width at half maximum (FWHM) after RTA [25,26,27]. However, there are no direct measurements of the improvement in the disorder after annealing treatment in dilute nitrides, probably due to the difficulty in spatially resolving the distribution of N [20,28]. In addition, the possibility of a synergistic effect in the case of GaAsSbN layers, by combining the presence of Sb and post-growth annealing, should not be ruled out.

This work has the goal to quantify and compare the compositional inhomogeneities of the N distribution in both type-I (GaAsSbN/GaAs) and type-II (GaAsSb/GaAsN) SL structures, before and after annealing. For this, we have used a methodology that is based on the treatment of Annular Dark Field (ADF) images in STEM mode to obtain N compositional mappings [29]. The homogeneity and uniformity of the N distribution is evaluated in terms of texture analysis, by gray level co-occurrence matrixes (GLCM) and the trend of clustering. In addition, the possible connection between regions with Sb and N clusters has been studied.

## 2. Materials and Methods

The SL samples under study were grown by solid source molecular beam epitaxy on GaAs (001) *n*^+^ substrates, under As_4_ overpressure conditions. Each sample consists of a 750 nm thick undoped active layer, grown at 470 °C, and at a rate of 1 monolayer per second (mL/s), deposited on a 250 nm thick, *n* doped GaAs buffer layer. Finally, the structure was capped with 50 nm of GaAs. The active region has periods of 40 mL of GaAsSbN/GaAs and GaAsSb/GaAsN in the SLI and SLII samples, respectively. Both samples have the same nominal flux for Sb (beam equivalent pressure of 2 × 10^−7^ Torr) and N (optical emission detection signal OED = 2.2 V). The average values of the N content by means of X-ray diffraction (XRD) simulations in the N-containing layers are 2.5% and 2.3% for SLI and SLII samples, respectively [30]. Pieces of both samples were subsequently annealed at 800 °C during 30 s under a nitrogen atmosphere using an RTA Mila 3000 ULVAC oven (ULVAC, (Methuen, MA, USA)). This temperature was chosen as the optimum amongst the three analyzed (750, 800, and 850 °C), meaning the one leading to a large increment in the PL peak and integrated intensity, and to a large reduction of the FWHM, while avoiding surface deterioration that starts at 850 °C. The annealed samples are named SLI_an_ and SLII_an_.

Cross-sectional samples were prepared using the lamella method, in a focused ion beam (FIB FEI Quanta 3D) (FEI Europe B. V., Eindhoven, The Netherlands). This TEM sample procedure permits the study and comparison of extended regions with similar TEM sample thicknesses. A structural and compositional analysis was performed by TEM. All of the samples with a thickness of about 100 nm were observed along the [110] direction. High angle and low angle annular dark field (HAADF and LAADF, respectively) studies and energy-dispersive X-ray (EDX) spectroscopy mapping were performed in STEM mode in a double-aberration-corrected FEI Titan3 Cubed Themis (FEI Europe B. V., Eindhoven, The Netherlands) operated at 200 kV. The acquisition was performed at an estimated convergence angle of 16 mrad, by using a camera length of 46 mm. ADF images were simultaneously acquired with two annular detectors: 30–126.2 mrad for LAADF images and 126.2–200 mrad for HAADF images. The EDX mapping was carried out with four embedded Bruker SDD detectors using ChemiSTEM technology and processed using Bruker’s ESPRIT software (1.9.4 version). EDX maps taken in different regions of the TEM samples was a figure of merit of the reproducibility found in the measurements and homogeneity of the growth. Where the GaAs substrate regions had a Sb content below 0.5%, that was considered the maximum error of the measurements. The analysis was completed by high-resolution X-ray powder diffraction (HR-XRD) in a Panalytical X´Pert Pro system (PANalytical, V. B. Almelo, The Netherlands) using the Cu Ka1 line (1.54056 Å). Taking into account the average Sb content measured by EDX, HR-XRD spectra allowed for the estimation of the average N composition, assuming a completely pseudomorphic epitaxy. The fitting was performed using X’Pert Epitaxy software by PANalytical. Once the fitting is completed, the N contents and layer thicknesses can be varied by ~0.1% N and 0.1 nm, while still maintaining a reasonable fit to the original spectrum.

## 3. Results and Discussion

**Figure 1:**
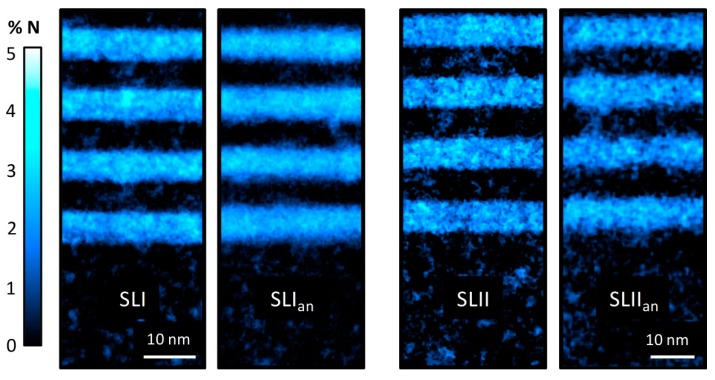
N compositional mappings for all samples along the [110] axis.

The analysis of Sb distribution in type-I and -II SLs have shown that Sb has been segregated strongly to the upper layers of GaAs(N), being present throughout the whole structure [31]. In fact, the incorporation of Sb is influenced by the presence of N, since, during the simultaneous growth with N in the GaAsSbN layers (SLI sample), a greater density of clustering appears with regard to the epitaxial deposition of only Sb atoms (GaAsSb layers in SLII sample) [30]. While the Sb distribution has been described earlier, the problem is that obtaining mappings of the N distribution it is more difficult. The reasons can be found in the very low N content in this type of alloy, making its characterization using common analytical techniques, such as EELS or EDX, a difficult task. Recently, the analysis during the simultaneous acquisition of LAADF and HAADF images at different detector inner angles in STEM mode allowed us to propose a method to obtain maps of the N distribution in SLs. Our method exploits the dependence of HAADF-STEM image intensities on the atomic number and static atomic displacements [32]. It is based on the appropriate image normalization, and separation of intensity coefficients in regions with and without Sb, known the mean content of N (see more details in [29]). The method provides images with intensities that are proportional to the N content. Using Sb contents from EDX mappings, it is possible to know the average N contents obtained from the XRD fitting. Calibrating the images intensity with these average data and following the procedure, N mappings have been plotted on both types of structures, before and after annealing, as shown in Figure 1. We use a look-up table (LUT) color scale, where white is related to the highest N content and black to zero. The mean N content in the substrate is less than 0.03% of N, which indicates the reliability of the procedure. However, the aim of this study is to evaluate the homogeneity of the N, therefore comparing the irregularities between the different regions of the layers. The standard deviation of the N content in the substrate region, always below 0.4%, should be considered as the precision of the method. The average thickness of the N-rich layers is narrower in the SLI sample (5.9 nm) than in the SLII sample (7.0 nm). A small difference in growth rate is the origin of the small discrepancies in the average layer thicknesses and compositions of both samples.

**Figure 2:**
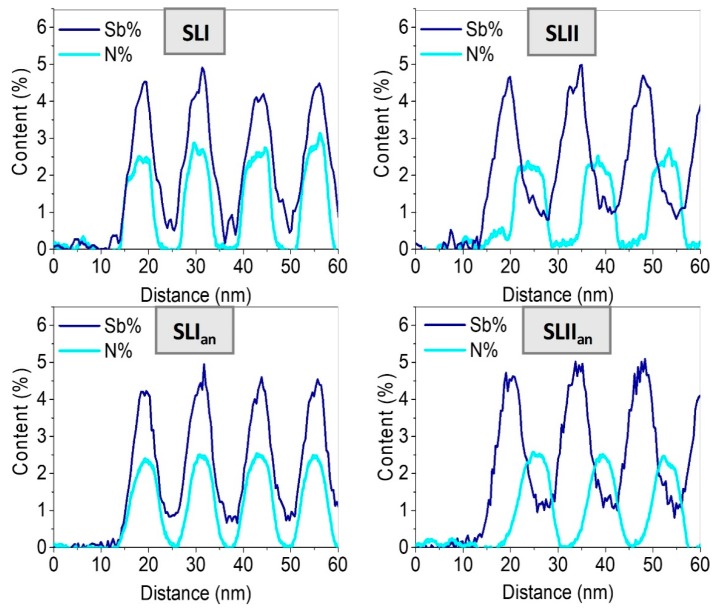
Average N and Sb profiles along the growth direction for all samples.

In these maps, a total absence of N is visible in the intermediate layers, in contrast to the case of Sb. Figure 2 shows the mean content profiles of N along the direction of growth for all samples, accompanied by the mean Sb profiles, obtained from EDX measurements. This set of data allowed us to appreciate the differences in interface quality. In the as-grown samples, the N profiles plot an almost square wave, with abrupt interfaces and a plateau with a constant composition. In contrast, Sb profiles take the form of a shark-fin wave, where the Sb content is always greater than zero along the entire structure. After annealing, all profiles are smoothed by thermal diffusion. The N-profiles take the shape of a Gauss curve, losing the abrupt character of the interfaces. In the case of SLI, the slight N gradient inside the layers disappears. In addition, the thicknesses of the N-containing layers after annealing are increased to 6.2 and 7.7 nm for the SLI_an_ and SLII_an_ samples, respectively. In the case of SLI_an_, despite the higher N composition, it undergoes the smallest increase in thickness with respect to the as-grown sample (6%) compared to the SLII_an_ sample, which marks 9%. It seems that the presence of Sb in the SLI_an_ could shorten the diffusion length of N, possibly due to a pinning effect of Sb to maintain local deformation compensation.

A simple visual exam of the images reveals that certain features, such as homogeneity or uniformity, are very different among them, especially after annealing. Recently, it has also been reported that GLCM is quite an effective methodology to identify these material features in microscopy images, especially when the structure is fairly homogeneous [33]. For sure, GLCM provides a mature and effective statistical method for analyzing textures and patterns in images. This effectiveness is because the method considers not only the distribution of intensities, but also the relative positions of pixels in the image [34]. In this work, several GLCM texture descriptors were evaluated: (i) inverse difference moment (IDM), which measures the smoothness (homogeneity) of the level distribution; (ii) correlation, which measures the linear dependency of gray levels on those of neighboring pixels; (iii) angular second moment (ASM), which measures the uniformity (or orderliness) of the gray level distribution; and (iv) entropy, which measures the degree of disorder among pixels. The results of applying the GLCM routines in the N-rich layers, in several images of every sample, are displayed in Table 1. First, a similar trend in all the parameters is observed, where the SLI samples are always more homogenous and uniform than the SLII ones. Second, there is an improvement of all the parameters after annealing, being higher in the SLI_an_ than in the SLII_an_ approach. Notably, the homogeneity in the N distribution is stronger in SLI than in SLII_an_.

**Figure 3:**
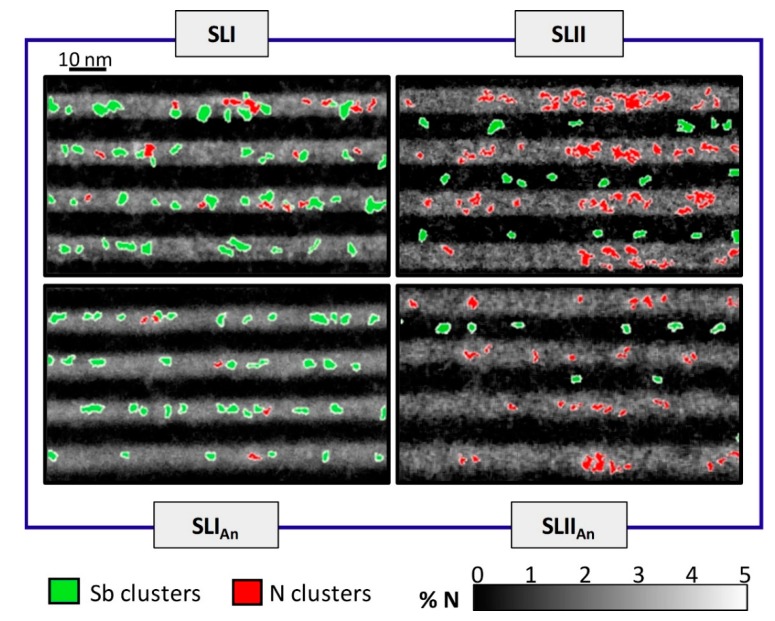
Grey-scale N mappings of all the structures, where the cluster areas of N and Sb are superimposed in red and green, respectively.

Another way to measure the homogenization of the distribution, in a sample, is assessing the degree of clustering. In the study, clusters have been considered as the regions with an N content 15% over the average, and with an area greater than 1.5 nm^2^. Additionally, this study has been carried out in particle areas, ranging from 0.75 to 2 nm^2^, obtaining similar trends. Figure 3 shows N maps of the different samples, where the positions of the N clusters are masked in red. A higher cluster density randomly distributed is observed in the type-II structure, with respect to type-I. It is important to note that the annealing treatment results in a significant reduction of clusters, in both types of SLs, i.e., the increase in diffusion leads to a better homogenization of the distribution of N within the layer. N is preferably reorganized within the GaAs(Sb)N layers, with a small diffusion towards the GaAs(Sb) barriers.

In order to correlate the distribution of N and Sb conglomerates, Sb composition maps from EDX measurements of the same region were simultaneously performed and used to locate regions richer in Sb, focusing on areas with Sb contents higher than 5.5%. Their position is superimposed in Figure 3 and masked in green. As can be deduced, the incorporation of Sb is influenced by the presence of N. Furthermore, a higher clustering density appears during simultaneous growth with N in the GaAsSbN layers (SLI sample), with respect to epitaxial deposition of Sb atoms only (GaAsSb layers in the SLII sample) [30]. In addition, annealing processes are demonstrated to result in greater Sb uniformity, along with improved interface roughness in both structures. Significantly, there is no correspondence between the distributions of the Sb and N conglomerates. This means that the values of the N-rich regions obtained in N maps are not affected by improper distinction of the Sb signal. It is important to note that this characteristic is contrary to expectations of an association between regions rich in Sb and N, in order to reduce the local stress. During the simultaneous incorporation of Sb and N on the growth front, it seems that they do not have a special preference to be accommodated together.

**Figure 4:**
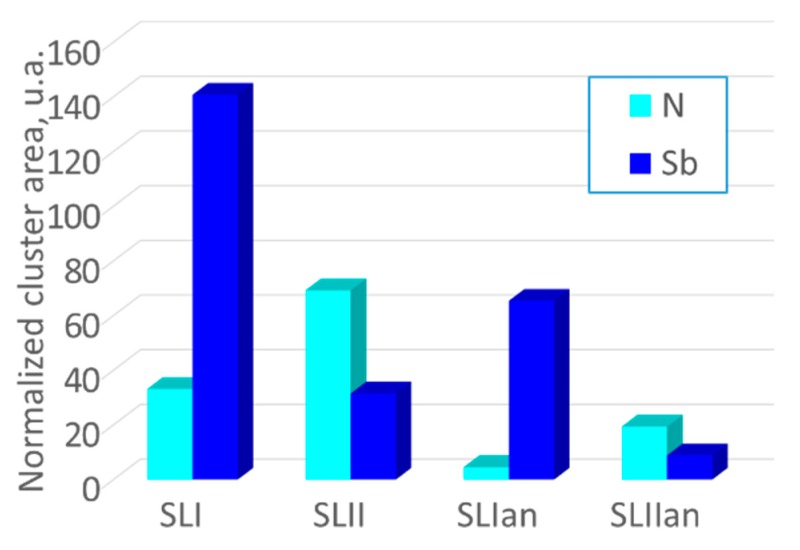
A bar graph showing the normalized cluster areas of Sb and N for all of the samples.

Figure 4 shows the normalized area for the N and Sb clusters, taking into account the differences in thicknesses, both the TEM samples, and the layers in every sample. As can be seen, the data concerning the area of the N clusters are in line with the GLCM results mentioned above. Certainly, the behavior is different when comparing the areas of the Sb- and N-rich regions between SLI and SLII configurations. The growth of GaAsSbN layers in SLI favors the agglomeration of Sb, but not N, which is more homogeneously incorporated. On the contrary, with the separation of N and Sb fluxes in the SLII sample, Sb has a lower tendency to aggregate in the GaAsSb layers and the opposite occurs with N in the GaAsN layer.

During the annealing, the reduction of the cluster area of N is higher in the SLI approach (86%) than in the SLII one (72%). However, the contrary occurs in the case of Sb, where the SLII sample undergoes a Sb cluster area decrease up to 70%, and the SLI sample undergoes a Sb cluster area decrease only up to 50%. N is incorporated more homogeneously in the presence of Sb, but at the expense of a greater agglomeration of Sb, compared to the growth of Sb alone. It is important to note that a post-growth annealing treatment further improves the homogeneity in those samples that were originally more homogeneous. The effect of post-growth treatments on these samples has recently been shown to have a great impact on solar cell performances [35], which should be related to the effects described in the study.

Taking all this into account, the more balanced sample is SLII_an_, and this result would explain the high increase in the PL intensity (up to 60%), regarding SLI_an_ designs [30]. Moreover, the type-II band alignment in these SLs provides long radiative lifetimes that may have potential advantages for carrier collection, which is not achievable in the type-I SLs [36]. All this explains why research into solar cell performance has focused on GaAsSbN type-II superlattices and the improvements that annealing treatments could make to these structures. Recently, the application of such an RTA process to GaAsSbN-based type-II solar cells has been shown that improves significantly the V_OC_, regarding the as-grown samples [35]. On the other hand, the experimental observation of a dramatic drop in carrier extraction efficiency from 6 nm to 12 nm period thickness in type-II SLs is explained by the transition in the dominant, electron, transport regime from quasi-ballistic to sequential tunneling accompanied by phonon-assisted thermal escape [11]. A further reduction in period thickness could improve the extraction efficiency, but we must be careful if we want to maintain the advantages of spatial separation of N and Sb, with respect to crystal quality and composition control. In addition, the advantages of the application of post-growth thermal annealing could be lost if the thickness of the period is in the order of the diffusion length of the species. However, although our results are very promising for the objective of integrating these structures as efficient sub-cells into MJSCs, more work is needed to obtain optimized designs in terms of compositions, period thicknesses, and annealing treatments.

## 4. Conclusions

In summary, the distribution of N in type-I (GaAsSbN/GaAs) and type-II (GaAsSb/GaAsN) SL structures before and after RTA are analyzed. The as-grown samples show abrupt interfaces regarding the N content, and a complete absence of N in the intermediate layers. After annealing, N-profiles move from an almost square wave to a Gauss curve shape, with a greater effect on the SLII sample. The evaluation of homogeneity by GLCM analysis and the trend to cluster formation shows that the simultaneous growth of Sb and N in the SLI approach leads to a more uniform distribution of N than in the SLII approach. However, the opposite occurs with respect to the Sb distribution. Annealing treatments result in a significant improvement in the homogenization of N and Sb distributions in both types of SLs, with SLII_an_ being the most balanced sample.

## Figures and Tables

**Table 1:** Values of the texture features for the N distribution in all the samples (a.u.).

	ASM (uniformity)	Correlation	IDM (homogeneity)	Entropy
SLI	0.71	2.29	238.3	7.82
SLI_an_	1.26	4.55	262.6	7.27
SLII	0.42	1.29	200.6	8.40
SLII_an_	0.60	1.84	215.4	7.96
